# Chefs’ Attitudes and Sensory Analysis of Invasive Crayfish (*Faxonius limosus*) Meat: Psychological and Culinary Aspects

**DOI:** 10.3390/foods14111898

**Published:** 2025-05-27

**Authors:** Maja Paunić, Jasmina Lazarević, Dubravka Škrobot, Ivana Čabarkapa, Stefan Šmugović, Milica Vidosavljević, Miloš Županjac

**Affiliations:** 1 Department of Geography, Tourism and Hotel Management, Faculty of Sciences, University of Novi Sad, 21000 Novi Sad, Serbia; stefan.smugovic@dgt.uns.ac.rs; 2 Institute of Food Technology, University of Novi Sad, 21000 Novi Sad, Serbia; jasmina.lazarevic@fins.uns.ac.rs (J.L.); dubravka.skrobot@fins.uns.ac.rs (D.Š.); ivana.cabarkapa@fins.uns.ac.rs (I.Č.); milica.popovic@fins.uns.ac.rs (M.V.); milos.zupanjac@fins.uns.ac.rs (M.Ž.)

**Keywords:** *Faxonius limosus*, sensory evaluation, perception of crayfish, attitudes of chefs

## Abstract

Considering the growing significance of sustainable gastronomy and the need for controlling the populations of invasive species, the aim of this study is to explore chefs’ attitudes toward the sensory and psychological aspects of using invasive crayfish meat (*Faxonius limosus*) from the Danube. The study was conducted using a survey questionnaire with a sample of 210 respondents, employing a consumption restriction scale based on various psychological aversions to non-traditional food sources. Binary logistic regression indicated a significant impact of psychological aversion on the likelihood of accepting this raw material. Thirty chefs participated in the sensory evaluation of the crayfish meat. The results revealed that the meat has potential for broad application in the preparation of gastronomic products.

## 1. Introduction

The spiny-cheek crayfish *Faxonius limosus* (Rafinesque, 1817) is a native species to the eastern part of North America. This species, introduced to Europe over a century ago, is now present in over 20 countries (Austria, Belgium, Bulgaria, Croatia, the Czech Republic, Denmark, Estonia, France, Germany, Hungary, Italy, Latvia, Lithuania, Luxembourg, the Netherlands, Poland, Romania, Slovakia, Slovenia, and Spain) and is considered as one of the most significant invasive aquatic species in European inland waters. Due to its invasive nature, it is classified as a species of general concern according to EU regulations on invasive alien species (IAS) [1,2,3,4,5].

The population of *F. limosus* is expanding along the Danube River basin [6,7]. It was first recorded in Serbia in 2002 near Apatin, and its presence has since been documented along the entire course of the Danube River and its tributaries [8]. The spread rate varies from 13 to 24.4 km per year, depending on ecological conditions [9]. Given its high expansion rate, it is assumed that the current invasive range of this species in Serbia is broader than previously known. The lack of focus for the management and cooperation further complicates its control of invasive alien species (IAS) [5,10].

Owing to its strong adaptability to various environmental conditions, it is capable of inhabiting a range of water bodies, including rivers, ponds, lakes, tributaries, and canals, with differing water qualities [11]. This omnivorous species feeds on aquatic plants, fish eggs, and invertebrates, posing a significant threat to biodiversity. It disrupts native ecosystems by predating on local species, consuming plant matter that forms critical habitats, and outcompeting native species for food and shelter.

The spiny-cheek crayfish shows several characteristics, such as strong adaptability, rapid maturation, short lifespan, high fecundity, and second mating period, which facilitate its fast population growth, giving it high invasive potential. Additionally, the negative impact of the spiny-cheek crayfish *F. limosus* on the native crayfish populations in Europe is expressed in competition for habitats, in which the invader is more adaptive, it is a carrier of crayfish plague, it is lethal for the European native crayfish, it can destabilize riverbanks, and it can modify other habitats, due to its burrowing behavior causing substantial economic damage [12]. Generally, the economic damage caused by invasive species could cost Europe billions of Euros per year and damage costs are continuing to rise [13].

The meat of this crayfish is a high-quality food product that is rich in protein (18–20%) and low in fat (0.14–1.69%), and it has a significant content of omega-3 fatty acids [10,14,15]. It is characterized by easy digestibility and low energy value (76 kcal) [14,15]. It is also a good source of minerals [16,17]. Despite its nutritional value, research on its sensory characteristics and applications in diet is limited [18]. Studies in the U.S. suggest that ecosystem preservation strategies could encourage its use as food [19].

However, consumers often reject such products due to cultural norms, aversion to invasive species, and psychological barriers [20,21,22]. Taste aversion—avoidance of certain tastes or types of food—further diminishes the likelihood of accepting invasive crayfish as a food source [23,24,25,26,27].

Aversion to new food products is associated with both personal and social factors. Personal factors include disgust toward new foods [28,29,30], while social factors are linked to cultural norms and circumstances [28,31,32]. Although there are recommendations to introduce invasive crayfish into the diet [19,33,34,35], the role of psychological factors in food acceptance and the potential for gastronomic preparation remains underexplored.

Studies in the EU mainly focus on algae and jellyfish, while invasive crayfish species remain neglected [36]. Research shows that food neophobia, unfamiliarity with the product, and a lack of awareness of sensory characteristics are major barriers to the acceptance of new food options. The majority of studies deal with insect consumption [25,27,37,38,39,40], while research on the sensory aspects of new food products is scarce [41].

Considering the aforementioned challenges and limitations, this research aims to investigate chefs’ attitudes toward the sensory and psychological aspects of using invasive river crayfish meat. After identifying these attitudes, a sensory evaluation of *Faxonius limosus* meat was conducted to determine its meat profile, acceptability, and recommendations for its use in gastronomic products.

## 2. Materials and Methods

### 2.1. Research Area

**Figure 1:**
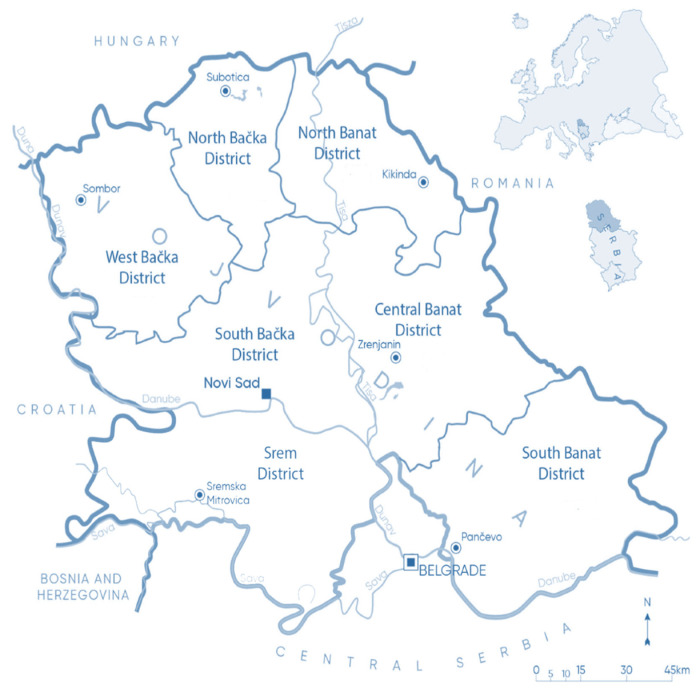
Research location (adapted and taken from Paunić et al.) [47].

The research area covers the territory of the Republic of Serbia, through which the Danube River flows over a distance of 588 km, making it the river with the longest course through a single country. Of this length, about 390 km flows through the Autonomous Province of Vojvodina [42]. For this reason, the area of Vojvodina was selected as the research site for this study (Figure 1). The crayfish specimens subject to sensory evaluation were captured near the area of Stari Slankamen. The samples were microbiologically safe, following the criteria defined by the European Commission Regulation No. 2073/2005 [43] on microbiological criteria for food and its amendments under Regulation (EC) No. 1441/2007 [44]. Microbiological safety of the evaluated samples was confirmed in a previous study by Lazarević et al. [10], titled “Invasive Crayfish *Faxonius limosus*: Meat Safety, Nutritional Quality and Sensory Profile”. 

Catering professionals involved in the survey were employed in catering establishments in the territory of Vojvodina, where they influence regional food preferences [45,46].

### 2.2. Research Design

Research exploring the attitudes of professional chefs toward the invasive river crayfish Faxonius limosus, as well as the sensory characteristics of this resource, is scarce and limited. Therefore, this study was designed and conducted in two phases: a survey and a sensory evaluation.

The questionnaire used in the research was developed in three stages: literature review, pilot testing, and final implementation.

It consists of three sections:

1. Sociodemographic data, including gender, age, and education level. These were collected using closed-ended questions with predefined answer options.
2. Psychological and sensory attitudes—Questions related to psychological and sensory perceptions were based on the Insect Phobia Scale (IPS), originally developed to measure fear and aversion to insects [25,27,48,49,50]. Its application was extended to cover fear and aversion toward unusual or unfamiliar food sources [51]. The original scale was adapted to suit the research topic and cultural context, in consultation with experts in gastronomy and hospitality. Responses were measured on a 5-point Likert scale (1 = strongly disagree; 5 = strongly agree).
3. Behavioral intentions—This section assessed participants’ willingness to use invasive crayfish meat in gastronomic products, based on the framework developed by Castro and Chambers [25]. The expressed attitudes allowed for an evaluation of the participants’ readiness to incorporate invasive crayfish meat into culinary practices.

Pilot testing was conducted in hospitality establishments across Vojvodina. The aim was to identify and eliminate potential ambiguities in question formulation. Participants were invited to comment on the clarity of the statements and mark any that were confusing or vague. Based on the feedback, minor adjustments were made to the wording and order of the items. The results indicated that the instrument was clear and comprehensible. In line with recommendations from the literature, the pilot sample size exceeded 10% of the planned main sample [52], involving 30 participants.

The study involved head chefs from various establishments. One representative from each establishment completed the survey. Participants were randomly selected based on probability sampling. The questionnaires were distributed electronically, based on the contact list of the National Association of Chefs of Serbia. Data collection occurred from 15 February to 15 April 2024. A total of 210 valid questionnaires were collected. All participants were first informed about the nature and the scope of the study, after which they gave their consent to participate voluntarily.

The second phase of the research was sensory profiling. Sensory analysis was carried out by a sensory panel consisting of thirty professional culinary chefs (7 females and 23 males, aged between 30 and 55 years). They had previously participated in the survey and were selected based on their expertise (at least three years of professional experience as chefs), knowledge, motivation, and availability. The chefs involved in the study were classified as semi-trained panelists, given their culinary expertise and prior experience in identifying, describing, and differentiating sensory attributes gained through formal university coursework. Culinary professionals cannot be considered sensory experts, but rather traditionally and experientially skilled in recognizing the sensory preferences and inclinations of their restaurant guests with respect to specific types of food [53].

Before the testing session, the panelists agreed on 32 descriptors related to crayfish meat that were used for sensory profiling. These are presented in Table 1.

The final set of descriptors was created through a consensus process, led by an experienced panel leader. This process included defining each descriptor and establishing assessment techniques. After the profiling session, panelists were asked to score the overall acceptability of each sample using a 9-point scale labeled on the left with 1 = “dislike very much” and on the right with 9 = “like very much.”

Finally, in the third phase of the Hedonic Analysis, panelists were asked to suggest how each sample could be used in the preparation of different types of meals and which type of wine would best complement the meal. The responses were grouped into categories: salad, pasta, risotto, appetizer, soup, main course, white wine, and red wine.

The samples, consisting of two pieces of crayfish meat each, were served individually in odorless plastic containers. Each sample was presented within 5 min of preparation and assigned a unique three-digit random code. A cup of water was made available to participants for palate cleansing. The evaluation occurred in individual sensory booths, each equipped with appropriate lighting, ventilation, and controlled temperatures. Participants were given written information about the study and signed an informed-consent form prior to their involvement.

The sensory evaluation of crayfish meat was conducted at the Accredited Sensory Laboratory of the Institute of Food Technology, University of Novi Sad, which was designed in compliance with international standards for sensory evaluation test rooms [54].

Descriptive statistics were used to present the basic characteristics of the sample (gender, age, and education level), including mean (M), standard deviation (SD), and frequencies expressed as percentages. This approach allows for a general overview of the sample and prepares data for further analysis [55]. Scale reliability was assessed using Cronbach’s alpha, with values above 0.7 considered acceptable and above 0.8 considered very good [56]. To compare attitudes between genders, an independent *t*-test was conducted, while one-way ANOVA was used to examine differences across age and education groups [57,58]. Post hoc tests were performed following significant ANOVA results [55]. Binary logistic regression was applied to predict positive or negative attitudes toward the consumption of invasive crayfish, identifying factors that significantly influence response likelihood [59].

Data processing was performed using the SPSS software program (version 24.0 for Windows). Data entry into the created matrix was systematic, followed by validity testing. Missing-data analysis was conducted within the missing-value analysis module. To assess the randomness of missing-data distribution, Little’s test was applied, which is the standard method for evaluating whether data are missing randomly or systematically [60]. This test is crucial to ensure that missing data do not affect the validity of the research results.

In sensory evaluation, results represent a mean value ± standard deviation of three individual measurements. The data obtained in the sensory analysis were submitted to principal component analysis (PCA), and the obtained sensory map was used to analyze the sensory profile and acceptability of crayfish. To explore the relationship between selected meal types and crayfish samples, correspondence analysis (CA) was conducted on the frequency table of qualitative data. One-way ANOVA was used to determine statistically significant differences (*p* < 0.05) across all variables, followed by Tukey’s multiple comparison test, using the XLSTAT 2018.7 software package (Addinsoft) for the statistical analysis.

### 2.3. Ethical Statement

Ethical approval was obtained from two relevant ethics committees: the Ethics Committee for the Protection and Welfare of Experimental Animals at the University of Novi Sad, Serbia (no. 04–81/94, EK: I–2020–06–1); and the Ethics Committee of the Scientific Institute of Food Technology in Novi Sad, University of Novi Sad (no. 175/I/22-3). According to national regulations (Annex 4 of the “Official Gazette of RS”, No. 39/10), ethical approval is not required for research involving invertebrate animals, as was formally stated in the ethical documentation.

The survey research was conducted anonymously and did not involve the collection of any personal data from the respondents. As such, this kind of research does not require special Ethical Committee approval in Serbia, where the research was conducted, as it is in line with the national Law on Personal Data Protection (The Official Gazette of the Republic of Serbia, number 97/08; hereinafter, the Law). The national Law on Personal Data Protection is aligned with the current standards of the relevant European documents and, in particular, with the EU General Data Protection Regulation (GDPR). The Law applies to the processing of personal data in the context of the activities of a controller or a processor in the Republic of Serbia, regardless of whether the processing takes place in the Republic of Serbia or not.

## 3. Results and Discussion

### 3.1. Presentation of Survey Results

A total of 210 respondents participated in the study, of which 60% were male and 40% were female. The average age was 36 years (SD = 10). The majority of respondents had completed secondary education (50%), followed by higher education (40%), while the smallest group had completed primary education (10%). The age structure of the respondents is consistent with previous related studies. While most of the previous research focused on the general consumer population [61,62,63,64,65,66], this study, based on recommendations from authors who have dealt with the attitudes of vegetarians, focused on a specific target group—chefs in the hospitality industry [61]. Their perception is particularly important because they actively shape food trends, influence consumer attitudes, and decide whether to accept new products as raw materials for gastronomic offerings [67,68].

The perception of chefs is of particular importance because they not only directly influence consumers’ culinary choices but also play an active role in creating and steering food trends [69,70]. As professionals who select and combine ingredients on a daily basis, they make decisions about incorporating new products into menus and establishing gastronomic standards. Their attitudes largely determine the potential acceptance of innovative yet less conventional ingredients [71]. This is why understanding their attitudes and willingness to experiment is crucial for the successful integration of invasive river crayfish into contemporary gastronomic offerings.

### 3.2. Descriptive Statistics and Reliability of the Scale

The results of the Crayfish Meat Consumption Restriction Scale in the preparation of gastronomic products are presented below. Six variables measure different aspects of psychological and sensory aversion to gastronomic products made from invasive river crayfish. The mean values and standard deviations for each variable are presented in Table 2. Cronbach’s alpha for the scale is 0.87, indicating satisfactory reliability.

The research results show strong negative attitudes toward the consumption of invasive river crayfish from the Danube among the participants. The average responses on a psychological and sensory attitudes scale from 1 to 5 indicate a high degree of disgust (M = 4.2, SD = 1.1) and concern regarding the sensory and food safety aspects of consuming these crayfish. This may represent a significant barrier to their wider inclusion in restaurant offerings [53]. 

It is important to pursue further research of this kind, as few studies have explored the attitudes of restaurateurs toward sustainable practices [72]. The most prominent concern expressed by participants was related to the food safety of gastronomic products made from invasive crayfish (M = 4.6, SD = 0.9). This finding may reflect a lack of information regarding the safety of invasive river crayfish [73], or a lack of trust in their safety, possibly linked to the perception of the Danube as a potentially polluted ecosystem [74]. Previous studies conducted in Vojvodina suggest that hospitality employees generally demonstrate a lower level of competence in food safety-related matters [75,76].

The results also reveal strong biases concerning the sensory attributes of invasive crayfish meat. Participants voiced concerns that dishes prepared with this species possess an unpleasant, muddy flavor (M = 4.5, SD = 1.0) and exhibit inferior sensory qualities compared to other types of crayfish (M = 4.4, SD = 1.1). These attitudes are not unexpected, given that most chefs have no prior experience working with this ingredient and likely formed their opinions based on assumptions [77]. Negative perceptions may also be influenced by the association of the Danube with pollution, leading to the belief that the crayfish meat absorbs undesirable aromas, particularly those associated with mud [75]. Additionally, the lower evaluation compared to other crayfish types may stem from the more common use of marine crayfish in restaurants, which are perceived as being higher in quality [78,79,80].

Beyond sensory and health-related concerns, participants also viewed the consumption of these crayfish as socially unacceptable (M = 3.8, SD = 1.3) and not aligned with local gastronomic culture and tradition (M = 4.3, SD = 1.2). These findings underscore the influence of cultural barriers that may inhibit the adoption of new food sources within restaurant offerings [81,82]. Such attitudes may be rooted in the absence of tradition regarding the use of river crayfish in local cuisine, as in some cultures, they are considered less prestigious than other seafood varieties [77].

### 3.3. Differences in Participants’ Attitudes Toward Invasive River Crayfish Based on Sociodemographic Characteristics

The results indicate that gender, age, and education significantly influence chefs’ attitudes toward invasive river crayfish, highlighting demographic differences that may affect their acceptance in gastronomy.

#### 3.3.1. Gender

The results of the *t*-test (Table 3) show a statistically significant difference between men and women (t = 2.4, *p* = 0.018), with women expressing significantly more negative attitudes (M = 4.4, SD = 1.1) compared to men (M = 3.9, SD = 1.0). Available studies suggest that women, particularly vegetarians, have shown greater acceptance of novel foods such as spirulina and seaweed [61,83]. However, other studies investigating the acceptance of new foods based on sociodemographic characteristics did not observe such differences [36]. It is important to note that studies in the field of food preferences often show that women tend to have stricter criteria when evaluating new or non-traditional foods, which could justify the findings of this study [84,85].

#### 3.3.2. Age Groups

The results of the ANOVA test (Table 4) indicate a statistically significant difference between age groups (F = 4.2, *p* = 0.007), with the youngest group (18–29 years) having the most positive attitudes (M = 3.8, SD = 1.2), while the oldest group (50+ years) holds the most negative views (M = 4.5, SD = 1.1). Post hoc analysis shows that the attitudes of respondents over 50 years old are significantly more negative compared to the youngest group.

These results suggest that younger chefs are more open to innovations and are potentially more inclined to experiment with non-traditional ingredients [86]. In contrast, older chefs may be more tied to traditional culinary norms [87], which may lead to greater resistance to incorporating invasive crayfish into restaurant menus.

#### 3.3.3. Education Level

The results of the ANOVA test (Table 5) show a significant difference in attitudes based on the education level of respondents (F = 5.3, *p* = 0.004), with the most negative attitudes found among respondents with basic education (M = 4.5, SD = 1.0), while those with higher education exhibit significantly more positive attitudes (M = 3.9, SD = 1.0). Post hoc analysis confirms that respondents with higher education have significantly more positive views compared to those with basic education.

These results suggest that higher education contributes to greater openness to new gastronomic concepts and experimentation with unconventional ingredients [88]. Additionally, respondents with higher education may be better informed about the ecological and economic aspects of using invasive species, which could contribute to their willingness to consider their use [89].

### 3.4. Regression Analysis

To examine the impact of the Scale of Restrictions on the Consumption of Crayfish Meat in the Preparation of Gastronomic Products, binary logistic regression was applied. The dependent variable was measured through “yes” and “no” responses. Out of 210 respondents, 42 (20%) expressed a positive attitude toward consuming invasive river crayfish, while 168 (80%) expressed a negative attitude.

The regression analysis (Table 6) revealed that all examined variables had a significant negative influence on respondents’ willingness to use invasive crayfish in dish preparation. The strongest effects were associated with perceptions of poor hygiene (B = −0.58, OR = 0.56, *p* < 0.001) and unpleasant taste (B = −0.52, OR = 0.60, *p* < 0.001), indicating that these are key factors in shaping negative attitudes. These findings are consistent with previous research emphasizing the role of hygiene and sensory characteristics—such as taste and texture—in the acceptance of novel food products [90,91,92].

Furthermore, the variables “idea of eating” (B = −0.45, OR = 0.64, *p* < 0.001) and “social acceptability” (B = −0.38, OR = 0.68, *p* = 0.006) were also significant predictors of negative attitudes toward the consumption of invasive crayfish. These results indicate that the acceptance of new food sources is shaped not only by individual preferences but also by broader societal factors [36,71,72]. The perception of social acceptability is particularly relevant in the hospitality context, where collective norms often influence ingredient selection and menu development [93,94].

In addition, the perception of crayfish as an inappropriate ingredient for gastronomic use (B = −0.41, OR = 0.66, *p* = 0.001) was significantly associated with psychological and sensory attitudes toward its use. This suggests that a lack of knowledge and experience in crayfish preparation may shape professional chefs’ perceptions [95,96]. The absence of crayfish in traditional cuisine and its limited availability both contribute to a cautious approach to its use, with gastronomic culture and tradition playing a central role in shaping attitudes toward the acceptance of novel food sources [97,98].

The findings also show that respondents generally express a negative attitude toward using invasive river crayfish from the Danube in the preparation of gastronomic products. Authors [99,100,101,102] emphasize the importance of educational campaigns aimed at reducing aversion and increasing openness toward novel ingredients. For this reason, the second part of the study included a sensory evaluation of crayfish meat, as taste is widely recognized as one of the key determinants in the acceptance of aquatic food products [64,66,103,104,105,106].

### 3.5. Sensory Evaluation of Crayfish Meat

**Figure 2:**
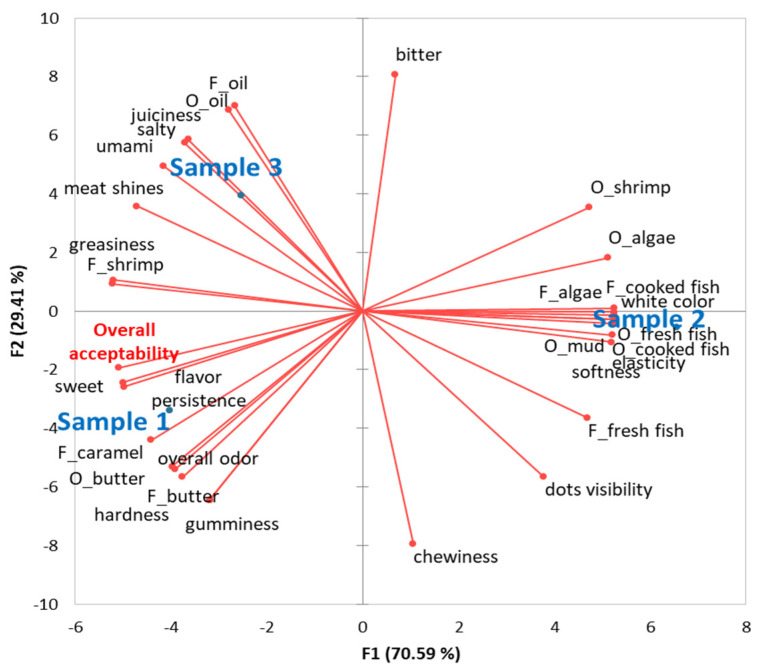
Principal component analysis was conducted to evaluate the sensory profile and acceptability of crayfish meat samples, including Sample 1 (fried in butter), Sample 2 (poached), and Sample 3 (roasted). Descriptor labels were coded with “O_” to indicate odor attributes and “F_” to indicate flavor attributes.

Figure 2 presents the sensory map illustrating the sensory profiles and overall acceptability of the crayfish meat samples. Samples were considered acceptable if their mean score for overall liking was above 5.0 (neither like nor dislike). Acceptability scores ranged from 4.96 for Sample 2 to 6.54 for Sample 1, indicating that the samples were rated as neutral to slightly liked.

The lower acceptability of Sample 2, prepared using the poaching technique, can be attributed to the more pronounced presence of algae-like odor and flavor, which are generally considered unpleasant and often associated with a muddy taste [107]. This result highlights the importance of preparation methods in reducing or enhancing specific sensory attributes, particularly when dealing with unfamiliar or less conventional protein sources.

In contrast, Sample 1, prepared by frying it in butter, achieved the highest acceptability score. This is likely due to the presence of familiar and pleasant sensory attributes—especially the odor and flavor of butter, caramel flavor, and a pronounced sweetness. These findings align with previous studies suggesting that positive and recognizable sensory traits significantly contribute to the acceptance of non-traditional foods [108].

The results demonstrate that the method of thermal processing plays a critical role in shaping the sensory perception and acceptability of invasive crayfish meat. This opens opportunities for further exploration of culinary techniques that may improve the overall experience of such ingredients and facilitate their integration into contemporary gastronomic offerings.

In the qualitative analysis, a total of 82 meal suggestions were collected and categorized into six main groups: main course, pasta, risotto, appetizer, salad, and soup. The proposed meal categories showed a statistically significant association with the analyzed crayfish meat samples (χ^2^ = 25.313, *p* < 0.05), suggesting that the sensory properties of the samples had a meaningful influence on the panelists’ culinary ideas and perceptions of appropriate food pairings.

**Figure 3:**
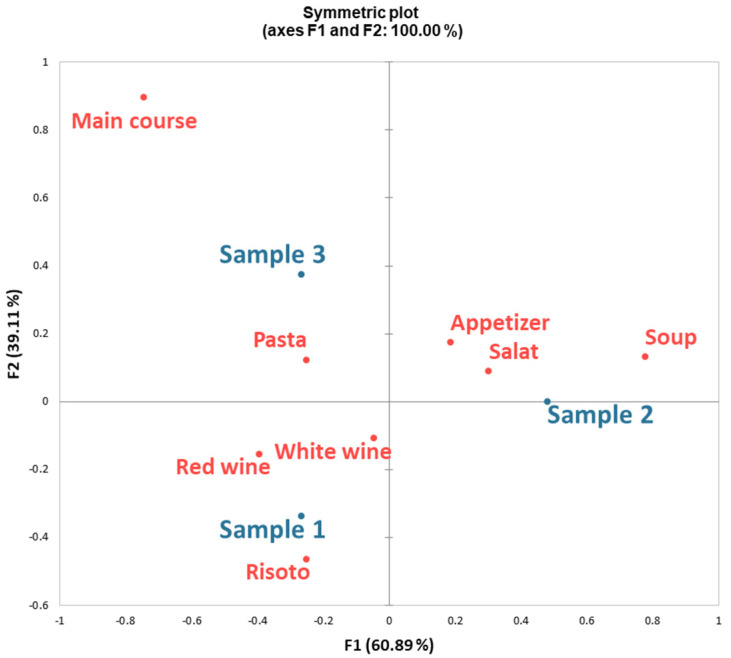
Correspondence analysis for crayfish samples and meal categories cited by professional culinary chefs.

To further explore these associations, correspondence analysis (CA) was conducted using the frequency of suggested meal types for each sample, with the results visualized in Figure 3. The analysis was presented in a two-dimensional factor plane, where the first two dimensions accounted for approximately 100% of the total variance, indicating a strong explanatory power of the model.

Interpretation of the CA map revealed clear trends in panelists’ preferences:

- Sample 1 (fried in butter) was predominantly associated with risotto and showed compatibility with both white and red wine. This may be due to its rich flavor profile and higher acceptability scores, as previously discussed.
- Sample 2 (poached) was more frequently suggested for appetizers, salads, and soups, indicating that its lighter sensory profile may be better suited for cold or delicately flavored dishes.
- Sample 3 (roasted) emerged as the most suitable option for pasta preparations, likely due to its more intense and robust flavor profile, which pairs well with starchy and sauce-based dishes.

These findings suggest that not only the acceptability, but also the perceived culinary applicability of invasive crayfish meat, depends largely on the chosen preparation method. The panelists, drawing from their professional experience, adapted their suggestions based on the sensory characteristics of each sample, reflecting a nuanced understanding of ingredient–function pairing in gastronomy [53]. Such results reinforce the importance of culinary context in shaping the acceptance and utilization of novel or underutilized food resources. They also emphasize the role of chefs as key mediators in the introduction of new ingredients into mainstream cuisine, particularly those that require careful handling or transformation to enhance their sensory appeal.

## 4. Conclusions

This study revealed predominantly negative attitudes among chefs toward the use of invasive crayfish from the Danube, primarily due to concerns regarding undesirable sensory characteristics and hygiene. Demographic factors such as gender, age, and education significantly influence these attitudes, with younger and more educated chefs showing a greater openness to innovation. Regression analysis indicates that the respondents’ willingness to prepare gastronomic products is influenced by their psychological and sensory perceptions.

Sensory analysis of crayfish meat samples showed varying levels of acceptability, which can significantly affect recommendations for its culinary use. This underscores the importance of understanding sensory attributes when incorporating invasive species into restaurant menus. Successful adaptation of crayfish-based dishes to their sensory profiles may increase acceptance and support wider use in gastronomy.

These findings may serve as a valuable source of inspiration for other regions and countries facing similar ecological and gastronomic challenges related to *Faxonius limosus* and other invasive species. By transforming ecological threats into culinary opportunities, the hospitality sector can contribute to environmental conservation and the development of gastronomic innovation.

In light of the results, future research should focus on developing standardized culinary techniques to enhance the sensory appeal of invasive crayfish, examining the impact of training and educational initiatives on chefs’ attitudes, and assessing consumer perceptions to determine the broader market potential of these species beyond professional kitchens.

## Figures and Tables

**Table 1:** The list of attributes used for sensory evaluation of crayfish meat.

Attributes	Descriptor	Definition	Technique
Appearance	White color nuance	Intensity of white color	Visually
	Meat shines	Glossiness that the surface of meat can have, typically influenced by moisture and fat contents	
	Dots visibility	Characteristic patterns on the crayfish meat	
Odor	Algae	Odor produced by algae	Olfactory
	Fresh fish	The distinct smell that fresh fish emits when it is freshly caught and properly handled	
	Cooked fish	The distinctive smell released when fish is prepared by cooking methods	
	Butter	The characteristic smell of butter, which can be described as creamy, rich	
	Oil	The smell emitted by various types of oils	
	Shrimp	The odor emitted by shrimp, which can range from slightly briny or seafood	
	Mud	The earthy smell produced by mud, typically described as damp, earthy, musty	
	Overall odor intensity	Overall odor intensity of the sample	
Taste	Sweet	Basic taste produced by dilute aqueous solutions of natural or artificial substances such as sucrose	Gustatory
	Salty	Basic taste produced by dilute aqueous solutions of various substances, such as sodium chloride	
	Bitter	Basic taste produced by dilute aqueous solutions of various substances, such as quinine or caffeine	
	Sour	Gustatory complex sensation, generally due to presence of organic acids	
	Umami	Basic taste produced by dilute aqueous solutions of a certain kind of amino acid	
Flavor	Algae	Flavor produced by algae	Olfactory, gustatory, and trigeminal sensations perceived during tasting
	Fresh fish	The distinct flavor that fresh fish emits when it is freshly caught and properly handled	
	Cooked fish	The distinctive flavor released when fish is prepared by cooking methods	
	Butter	The characteristic flavor of butter, which can be described as creamy, rich	
	Oil	The flavor emitted by various types of oils	
	Shrimp	The flavor emitted by shrimp, ranging from slightly briny to seafood	
	Caramel	The rich flavor that originates from the process of caramelization	
	Flavor persistence	The length of time a specific flavor lingers in the mouth	
Texture	Hardness	The force required to achieve a given deformation	The begins during mastication (chewing) and is further assessed during the swallowing process.
	Elasticity	The degree to which a deformed material returns to its original condition after the deforming force is removed	
	Gumminess	The effort required to disintegrate the product to the state ready for swallowing	
	Softness	The ease with which a food deforms under slight pressure or force	
	Juiciness	The amount and release of liquid from a food product during chewing	
	Chewiness	The work required to masticate a solid product into a state ready for swallowing	
	Greasiness	The quantity or the quality of fat on the surface	

**Table 2:** Mean values and standard deviations for each variable—Crayfish Meat Consumption Restriction Scale—in the preparation of gastronomic products.

Variable	Mean (M)	SD
The idea of eating invasive crayfish from the Danube River causes me disgust/aversion.	4.2	1.1
Consumption of invasive river crayfish from the Danube is not socially acceptable.	3.8	1.3
I am afraid that food based on invasive river crayfish has an unpleasant taste resembling mud.	4.5	1.0
I think that gastronomic products made from invasive river crayfish have worse sensory characteristics.	4.4	1.1
I believe that dishes prepared from invasive river crayfish are not safe for health.	4.6	0.9
I think that the use of invasive river crayfish from the Danube is not characteristic of our gastronomic culture and tradition.	4.3	1.2

**Table 3:** Gender differences in perception of limitations in crayfish meat consumption for culinary products.

Variable	Men (M ± SD)	Women (M ± SD)	*t*-test (t, *p*)
Disgust toward consumption of invasive crayfish	3.9 ± 1.0	4.4 ± 1.1	t = 2.4, *p* = 0.018
Health safety of crayfish	4.2 ± 1.0	4.6 ± 0.9	t = 2.1, *p* = 0.040
Sensory characteristics of crayfish meat	4.0 ± 1.1	4.5 ± 1.0	t = 2.3, *p* = 0.021
Social acceptability	3.7 ± 1.2	4.1 ± 1.3	t = 1.9, *p* = 0.060
Tradition in gastronomy	3.9 ± 1.1	4.3 ± 1.2	t = 2.0, *p* = 0.050

**Table 4:** Differences between age groups regarding limitations in crayfish meat consumption for culinary products.

Age Group	Mean (M)	SD	F	*p*-Value	Post Hoc Differences
18–29 years	3.8	1.2	4.2	0.007	18–29 < 50+
30–49 years	4.2	1.0			
50+ years	4.5	1.1			

**Table 5:** Differences between education levels regarding limitations in crayfish meat consumption for culinary products.

Education Level	Mean (M)	SD	F	*p*-Value	Post Hoc Differences
Basic	4.5	1.0	5.3	0.004	Higher < Basic
Secondary	4.3	1.1			
Higher	4.5	1.1			

**Table 6:** Regression analysis.

Variable	B	SE	Wald	*p*-Value	OR	95% CI
Idea of eating	−0.45	0.12	14.2	0.00	0.64	0.51–0.79
Social acceptability	−0.38	0.14	7.4	0.006	0.68	0.52–0.88
Unpleasant taste	−0.52	0.11	21.8	0.000	0.60	0.48–0.74
Unpleasant consistency	−0.49	0.13	14.0	0.000	0.61	0.48–0.78
Poor hygiene	−0.58	0.10	33.6	0.000	0.56	0.45–0.69
Inappropriateness	−0.41	0.13	10.1	0.001	0.66	0.52–0.84

## Data Availability

The original contributions presented in the study are included in the article, further inquiries can be directed to the corresponding author.
